# Incidence of Diabetes Mellitus and Obesity and the Overlap of Comorbidities in HIV+ Hispanics Initiating Antiretroviral Therapy

**DOI:** 10.1371/journal.pone.0160797

**Published:** 2016-08-10

**Authors:** Angelina Gomes, Emily V. Reyes, L. Sergio Garduno, Rita Rojas, Geraldine Mir Mesejo, Eliza Del Rosario, Lina Jose, Carmen Javier, Catherine Vaughan, Yeycy Donastorg, Scott Hammer, Karen Brudney, Barbara S. Taylor

**Affiliations:** 1 Department of Medicine, Columbia University Medical Center, New York, New York, United States of America; 2 Unidad de Tratamiento de ITS y VIH, Instituto Dermatológico y Cirugía de Piel “Dr. Humberto Bogaert Diaz”, Santo Domingo, Dominican Republic; 3 University of Texas Health Science Center San Antonio, San Antonio, Texas, United States of America; 4 Departamento de Medicina, Profamilia, Santo Domingo, Dominican Republic; 5 Unidad de Vacunas, Instituto Dermatológico y Cirugía de Piel “Dr. Humberto Bogaert Diaz”, Santo Domingo, Dominican Republic; Rush University, UNITED STATES

## Abstract

**Background:**

Cardiovascular disease (CVD) is a leading health threat for HIV+ patients on antiretroviral therapy (ART); cardiometabolic comorbidities are key predictors of risk. Data are limited on incidence of metabolic comorbidities in HIV+ individuals initiating ART in low and middle income countries (LMICs), particularly for Hispanics. We examined incidence of diabetes and obesity in a prospective cohort of those initiating ART in the Dominican Republic.

**Methods:**

Participants ≥18 years, initiating ART <90 days prior to study enrollment, were examined for incidence of impaired fasting glucose (IFG), diabetes mellitus (DM), overweight, and obesity. Fasting plasma glucose (FPG) 100-125mg/dl defined IFG; FPG ≥126 mg/dl, diagnosis per medical record, or use of hypoglycemic medication defined DM. Overweight and obesity were BMI 25–30 and ≥30kg/m^2^, respectively. Dyslipidemia was total cholesterol ≥240mg/dl or use of lipid-lowering medication. Framingham risk equation was used to determine ten-year CVD risk at the end of observation.

**Results:**

Of 153 initiating ART, 8 (6%) had DM and 23 (16%) had IFG at baseline, 6 developed DM (28/1000 person-years follow up [PYFU]) and 46 developed IFG (329/1000 PYFU). At baseline, 24 (18%) were obese and 36 (27%) were overweight, 15 became obese (69/1000 PYFU) and 22 became overweight (163/1000 PYFU). Median observation periods for the diabetes and obesity analyses were 23.5 months and 24.3 months, respectively. Increased CVD risk (≥10% 10-year Framingham risk score) was present for 13% of the cohort; 79% of the cohort had ≥1 cardiometabolic comorbidity, 48% had ≥2, and 13% had all three.

**Conclusions:**

In this Hispanic cohort in an LMIC, incidences of IFG/DM and overweight/obesity were similar to or higher than that found in high income countries, and cardiometabolic disorders affected three-quarters of those initiating ART. Care models incorporating cardiovascular risk reduction into HIV treatment programs are needed to prevent CVD-associated mortality in this vulnerable population.

## Introduction

The advent of antiretroviral therapy (ART) has greatly increased life expectancy for people living with HIV (HIV+),[1] but cardiovascular disease (CVD) is now a leading health threat in patients receiving ART.[2–5] HIV infection confers increased risk for CVD,[3, 6] and is linked to ART-induced dyslipidemia,[7–9] the chronic inflammation induced by HIV infection,[3, 10, 11] and conventional risk factors for CVD, like increasing age, minority race, smoking, dyslipidemia, and obesity.[4, 12–15] As HIV+ individuals’ lifespans approach that of the general population, the management of modifiable cardiovascular risk factors becomes increasingly important.

Although research in low and middle income countries (LMICs) has demonstrated increasing prevalence of diabetes and obesity in HIV+ individuals initiating antiretroviral therapy,[16–20] little is known regarding the incidence of obesity in HIV+ individuals initiating antiretroviral therapy in Latin America, a region heavily impacted by the global obesity epidemic. The incidence of diabetes has been measured in only two studies in LMICs, and few data exist on the incidence of diabetes in HIV+ individuals living in Latin America. Given the known increased risk of metabolic disorders such as diabetes and obesity in HIV-uninfected Hispanics in the United States (U.S.),[21–23] Hispanics in LMICs may be at high risk for developing metabolic complications after ART initiation.

The Dominican Republic (DR) is an ideal setting in which to examine the incidence of cardiometabolic complications after ART initiation. It is a resource-constrained country in the Caribbean with a high burden of obesity (26.6%), diabetes mellitus (9.9%), and dyslipidemia (12.3%),[24] and CVD is the leading cause of death (35%).[25] The Dominican national HIV treatment program currently provides ART to 48% of people in need of treatment,[26] ensuring a near normal lifespan for these individuals. However, this success could be jeopardized by HIV’s long-term cardiometabolic complications in a population already highly impacted by CVD.

We hypothesized that the known baseline increased risk of obesity and diabetes for HIV uninfected Hispanics in the U.S. could lead to increased risk of these conditions in HIV+ Hispanics receiving care in LMICs. We determine the incidence and overlap of key CVD risk factors: diabetes mellitus (DM), impaired fasting glucose (IFG), obesity, and overweight BMI in a longitudinal, prospective cohort of HIV+ patients initiating ART. We also examined the overall CVD risk and prevalence of cardiometabolic comorbidities. Our findings may have implications for ART expansion in LMICs, as cardiometabolic comorbidities would reduce the improvements in life expectancy offered by effective HIV treatment.

## Methods

### Study Patients

Patients included in this analysis participated in the Dominican HIV Cohort, a prospective, observational cohort study of HIV treatment outcomes in the DR from September 1, 2007 through May 31, 2013. Continuous enrollment took place at two adult HIV treatment centers in Santo Domingo, Dominican Republic, participating in the Dominican national ART program. Physicians and patient educators in the clinic offered enrollment to individuals initiating or already receiving ART. Of those offered enrollment, approximately 75% accepted. The primary reasons for refusal were work conflicts or living at a distance from the clinic. The cohort population was representative of the general clinic population.

**Fig 1:**
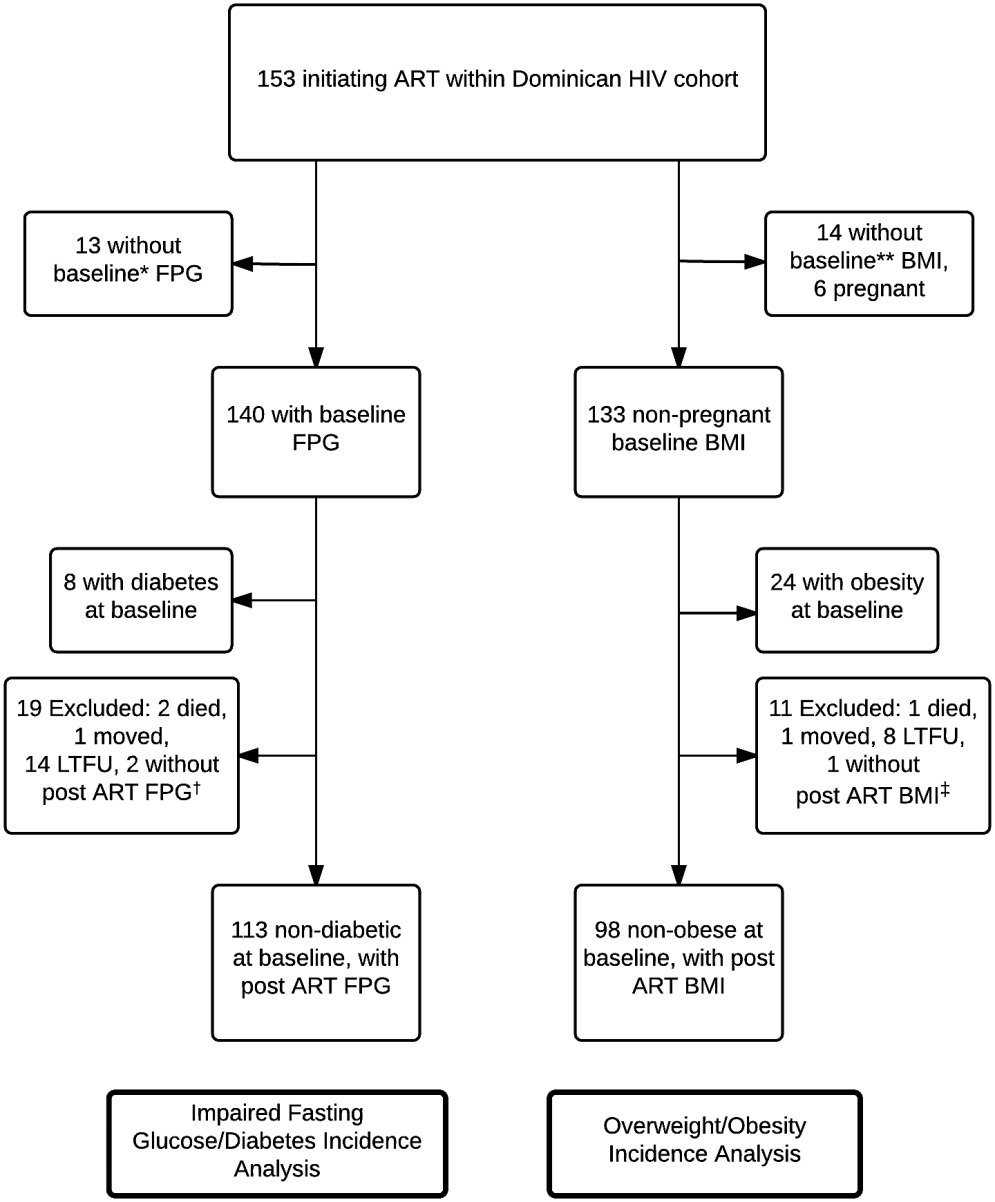
Distribution of cohort patients between impaired fasting glucose/diabetes and overweight/obesity analyses. ART: antiretroviral therapy; BMI: body mass index; FPG: fasting plasma glucose; LFTU: lost to follow up. *Baseline for DM analysis defined as ≤180 days prior to ART start date. **Baseline for obesity analysis defined as BMI measured ≤180 days prior to ART start date or up to 30 days after ART start. †Post ART FPG measured ≥2 weeks after ART initiation. ‡Post ART BMI measured ≥180 days after ART initiation.

Patients were included in this study if they initiated combination ART within 90 days prior to or any time after the first study visit. Patients were excluded if they had previously taken combination ART. Study patients were included in the analysis of DM and IFG if they had a baseline fasting plasma glucose (FPG) measurement ≤180 days prior to ART start date, and a second measurement ≥2 weeks after ART initiation. Non-pregnant study patients were included in the analysis of obesity and overweight BMI if measurements of height and weight were taken ≤180 days prior to or ≤30 days after ART start, and again ≥180 days after ART initiation. The distribution of cohort patients in the two analyses is represented in Fig 1. Participants were followed throughout the observation period with visits every 3 months in the first year of participation and every 6 months thereafter.

### Outcomes

Our primary outcomes of interest for this study were incidence of: (1) DM or IFG, and (2) obesity or overweight BMI. DM was defined as at least one FPG ≥126 mg/dl, documented use of hypoglycemic medication, or DM diagnosis per the medical record, as in other studies in LMICs and other settings where confirmatory testing is not always available.[27–29] IFG was defined as at least one FPG ≥ 100 mg/dl and no FPG ≥126 mg/dl. DM observation endpoint was the first date at which patients met criteria for DM, or the date of last FPG if patients did not meet criteria for DM. Eight individuals who were determined to have DM at baseline were excluded from further DM-related analyses described below.

Obesity was defined as BMI ≥30 kg/m^2^ and overweight as BMI ≥25 kg/m^2^. Observation endpoint dates were the first date when patients met criteria for obesity, or the date of the last BMI if patients never met criteria for obesity. Twenty-four patients who were obese at baseline were excluded from the incidence analyses.

### Covariates

#### Demographics

Demographic variables included gender, age at the beginning of the observation period, and HIV transmission categories. Self-reported transmission categories were categorized as heterosexual sex, men who have sex with men (MSM), and other, including unknown or missing. No patients reported maternal to child transmission or injection drug use as HIV transmission risk.

#### HIV disease parameters

We calculated the years since HIV diagnosis at the beginning of the observation period, and the duration of HIV diagnosis from diagnosis to the endpoints of our primary outcomes. Immunologic status was categorized, based on CD4 count taken ≤180 days prior to and closest to ART start date (hereafter baseline CD4 count), as <200 or ≥200 cells per microliter. Baseline viral load status, based on HIV-1 RNA assay taken ≤180 days prior to and closest to ART start date, was unavailable for most of the cohort because pre-ART testing is not offered within the Dominican national program, but when available, was categorized as <1000 or ≥1000. Endpoint immunologic and virologic status were categorized, based on CD4 counts and HIV-1 plasma RNA assays measured within ±180 days from each endpoint, as <200 or ≥200 cells per microliter and <400 or ≥400 copies per milliliter, respectively. Exposure to ART, defined as the use of ≥2 NRTIs (stavudine, lamivudine, emtricitabine, zidovudine, tenofovir, or abacavir) with ≥1 PI (lopinavir/ritonavir) or 1 NNRTI (nevirapine or efavirenz), was calculated in months from the ART initiation date to each outcome endpoint. These were the only combinations of ART available, as first and second line treatment, through the Dominican national HIV treatment program. Months on PIs and NNRTIs were calculated from treatment initiation date of the drug class to each outcome endpoint.

#### Other clinical variables

Dyslipidemia was defined as ≥1 measurement of total cholesterol ≥240 mg/dl, per the National Cholesterol Education Program (NCEP ATP III) guidelines,[30] or documented use of lipid lowering medication in the presence of normal serum cholesterol levels. As statins are relatively inaccessible due to cost in the DR, we included statins, fibrates, cholesterol absorption inhibitors, niacin, and bile acid resins as lipid-lowering therapy. Hypertension was defined as ≥2 blood pressure measurements with systolic blood pressure ≥140 or diastolic blood pressure ≥90, as per the Joint National Committee seventh report guidelines, or as the use of anti-hypertensive medications as documented in the medical record.[31]

Patients were asked about use of tobacco, alcohol, and drug use every 6 months. Tobacco users were classified as current users or not current users, as per the Framingham CVD equation. Substance users were categorized as current users or non-current users throughout the observation period. Alcohol use was categorized as average number of drinks consumed per month over the observation period. Depression was defined as any positive response during the observation period to the Patient Health Questionnaire-2 (PHQ-2),[32] administered at each cohort visit.

Ten-year CVD risk scores were calculated at the end of observation with the Framingham CVD equation for those patients aged 30–74.[33, 34] Patients were included in calculations if their last HDL measurement was taken ≥30 days after ART initiation, they had a blood pressure measurement taken within 1 year of their last HDL measurement, and had no reported history of CVD. Overall prevalence of cardiometabolic multi-morbidity was assessed by determining the overlapping prevalence of 1) IFG/DM, 2) overweight/obesity, and 3) dyslipidemia.

### Statistical Analysis

Bivariate analysis assessed associations between outcomes and the covariates above. Pearson Chi-square tests or Fisher’s Exact test tested associations between categorical variables. All continuous variables were non-normally distributed, so Mann-Whitney U tests determined differences in medians of continuous covariates by outcome. Survival analysis with Kaplan-Meier curves described incidence of DM, IFG, obesity, and overweight during the observation period. All analyses conducted 2-sided, with a significance cutoff of p<0.05 in IBM SPSS Statistics version 22 (Armonk, New York) and R version 3.1.0 (R Foundation for Statistical Computing, Vienna, Austria).

### Ethical considerations and funding source

Patients provided written informed consent prior to participating. The study protocol was approved by the Dominican National Committee on Bioethics (CONABIOS), the ethics committees of the Instituto Dermatológico y Cirugía de Piel “Dr. Huberto Bogaert Diaz” and Profamilia, and the Institutional Review Boards at Columbia University Medical Center and University of Texas Health Science Center at San Antonio.

## Results

Of patients initiating ART within the cohort in 2007–2013, 140 met inclusion criteria for the fasting glucose analysis and 133 met inclusion criteria for the BMI analysis (Fig 1). The median observation period was 34.8 months. The cohort was 57% female, with a median age of 39 (33–45), a median duration of HIV diagnosis of 3 years (IQR: 0–7), and heterosexual transmission was the dominant HIV risk factor (Table 1).

**Table 1:** Baseline characteristics of participants.

DEMOGRAPHIC CHARACTERISTICS	N (%)*
Total No. Patients	153
Women^1^	87 (57%)
Median age at baseline^2^ in years (IQR)	39 (33–45)
HIV transmission risk category^3^	
*Heterosexual*	138 (90%)
*MSM*	7 (5%)
*Other (work exposure*, *blood transfusion*, *unknown)*	9 (6%)
**HIV DISEASE PARAMETERS**	
Median duration of HIV in years at baseline^2^ (IQR)	3 (0–7)
Baseline^2^ CD4 <200 cells/μL (of 145 available)	66 (46%)
Median baseline^2^ CD4 (cells/μL) (IQR)	211 (135–265)
Baseline^2^ VL <1000 copies/mL (of 58 available)	4 (7%)
Median baseline^2^ VL (copies/mL) (IQR)	74,241 (18,784–178,5804)
Initiated ART with PI-based regimen	5 (3%)
Initiated ART with NNRTI-based regimen	148 (97%)
**CARDIOVASCULAR RISK FACTORS**	
Median baseline BMI^4^ (kg/m^2^) (IQR)	24.5 (22–28)
Hypertension^5^	30(20%)
Lipid lowering medication use	22 (14%)
Current smoker	21 (14%)
**OTHER RELEVANT CLINICAL CHARACTERISTICS**	
Depressive symptoms during observation period	126 (82%)
Current drug use (heroin, crack/cocaine, marijuana, or ecstasy)	9 (6%)
Average # drinks consumed per month throughout observation period	
*0*	58 (38%)
*1–10*	81 (53%)
*≥11*	14 (9%)

* Percent unless otherwise specified

^1^ No patients identified as transgender in this cohort

^2^Baseline measures taken ≤180 days prior to ART initiation

^3^ No patients reported transmission risk factors of intravenous drug use or vertical transmission

^4^Body mass index, baseline measures taken ≤180 days prior to ART initiation or ≥30 days after ART initiation

^5^ Hypertension was defined as systolic blood pressure ≥140 or diastolic blood pressure ≥90

### Incidence of impaired fasting glucose and diabetes mellitus

**Fig 2:**
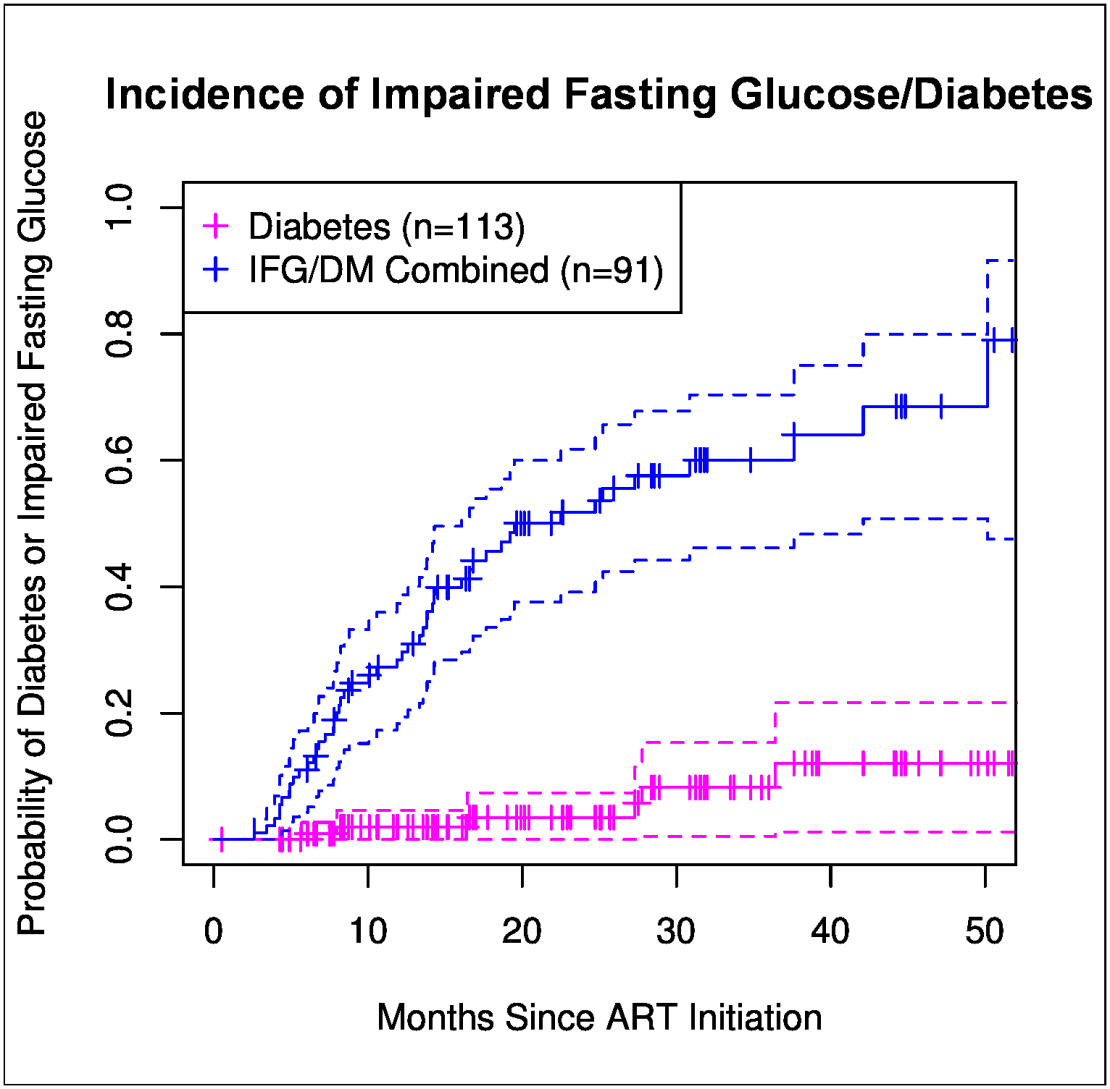
Kaplan-Meier curves representing risk of developing impaired fasting glucose /diabetes. Pink line represents the risk of developing diabetes (DM) and blue line represents risk of the combined endpoint of impaired fasting glucose (IFG)/DM, from normoglycemic baseline as a function of the number of months of ART. DM defined as ≥1 fasting plasma glucose (FPG) ≥126, documented use of hypoglycemic medication, or DM diagnosis per the medical record. IFG defined as ≥1 FPG ≥ 100 mg/dl and no FPG ≥126 mg/dl. Vertical tick-marks indicate patients whose survival times have been right-censored. Dashed lines represent 95% confidence intervals.

Of 140 patients in the diabetes analysis, 8 (6%) had DM and 23 (16%) had IFG at baseline, defined henceforth as ≤180 days prior to starting ART. After ART initiation, 6 of 113 (5%) patients with either normoglycemic or IFG measurements at baseline developed DM, resulting in an incidence of 28 per 1000 PYFU (Fig 2). Forty-six of 91 (51%) patients who were normoglycemic at baseline developed IFG, resulting in an incidence of 329 per 1000 PYFU. Patients with IFG had received ART for a median duration of 24 months (IQR: 13–39) when they met endpoint criteria. Fifteen (13%) patients received a PI containing regimen over the course of the observation period, but PI use was not statistically significantly associated with development of IFG or DM (p = 0.424). Those with the combined endpoint of incident IFG/DM were more likely to have dyslipidemia (OR, 8.23, 95% CI: 2.23 to 30.4). No other covariates, including baseline or endpoint CD4 count and HIV RNA levels, were statistically significant predictors of incident DM or IFG. The median observation period for patients included in the DM analysis was 23.5 months (IQR: 16.1–37.9). At the end of observation, a total of 14 (10%) and 69 (49%) patients met criteria for DM and IFG, respectively.

### Incidence of overweight and obesity

**Fig 3:**
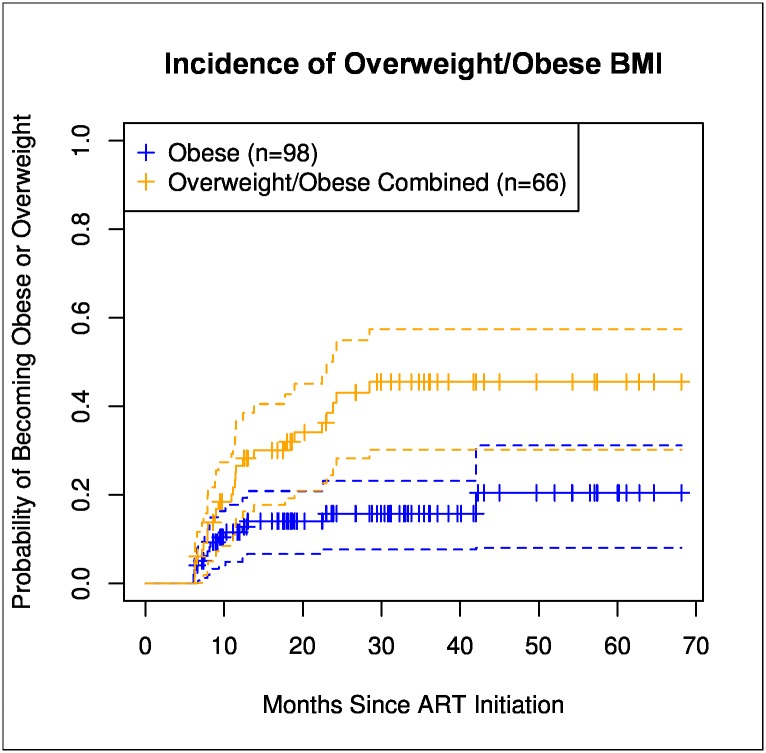
Kaplan-Meier curves representing risk of developing overweight/obese BMI. Blue line represents risk of developing obesity and orange line represents risk of the combined endpoint of overweight/obese BMI from normal BMI at baseline as a function of the number of months of ART. Obesity was defined as BMI ≥30 kg/m^2^ and overweight as BMI ≥25 kg/m^2^. Vertical tick-marks indicate patients whose survival times have been right-censored. Dashed lines represent 95% confidence intervals.

Of 133 patients in the obesity analysis, 24 (18%) were obese and 36 (27%) were overweight at baseline. At least 6 months after initiating ART, 15 (15%) patients became obese from normal or overweight BMIs, resulting in an incidence of 69 per 1000 PYFU (Fig 3). Twenty-two (33%) patients became overweight from normal baseline BMI, resulting in an incidence of 163 per 1000 PYFU. Incident obesity after ART initiation was associated with: age over 50 years (OR 9.88; 95%CI: 2.27, 42.9), and dyslipidemia (OR, 2.72; 95%CI: 1.20, 6.17). No statistically significant association with duration of PI use, baseline or endpoint CD4 count, or HIV RNA levels were observed. The median observation period for patients included in the obesity analysis was 24.3 months (IQR: 15.4–39.5). Patients with incident obesity had received ART for a median duration of 8 months (IQR: 7–12) when they developed BMI≥30 kg/m^2^. Few patients lost weight after becoming obese or overweight; eight patients went from obese to overweight, no obese patients reached a normal BMI (<25 kg/m^2^), and ten patients went from overweight to a normal BMI by the end of observation. However, whether this weight loss was intentional is difficult to determine. Considering changes in weight, at the end of observation, 24% of the cohort was obese and 42% was overweight.

### Burden of Cardiometabolic Risk

Framingham ten-year year risk scores were calculated for the 82 patients >30 years of age, with a median age of 42.4 (IQR: 38.2–50.6) at the time of score calculation. Two (2%) patients were classified as high risk (>20%), and nine (11%) were at moderate risk (10–20%). Two individuals suffered cardiovascular events during the observation period and were excluded from risk calculations. Thirty (20%) patients met criteria for hypertension, twenty (67%) of whom were receiving anti-hypertensive medication. Of those receiving treatment, seven (35%) patients had uncontrolled blood pressure, >140/90 (or 130/80 for diabetics) at their last measurement.

**Fig 4:**
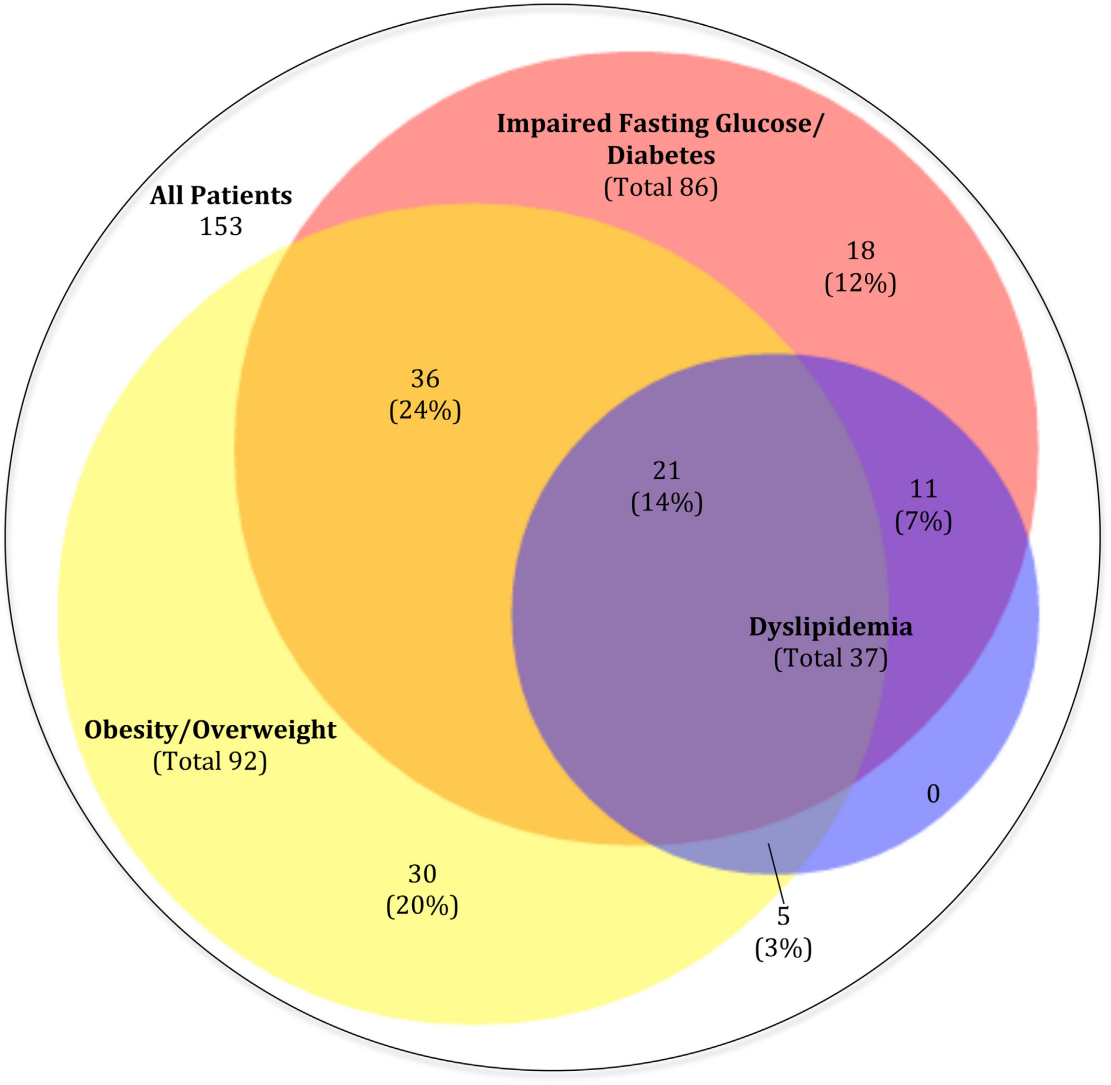
Burden of cardiometabolic comorbidities. Venn diagram representing the burden of overlapping cardiometabolic morbidity within the cohort of patients initiating antiretroviral therapy, depicting the combined endpoints of impaired fasting glucose/diabetes mellitus (pink), overweight/obesity (yellow), and dyslipidemia (blue), and the percentage of overlapping prevalence of these disorders. Definitions of these outcomes can be found in methods section.

Thirty-five (24%) of 143 patients who had lipid panels measured during observation had dyslipidemia based on serum lipid measurements or use of lipid-lowering medications. Analysis of the overall prevalence of cardiometabolic multi-morbidity (IFG/DM, overweight/obesity, or dyslipidemia) revealed 79% of the cohort had at least one cardiometabolic comorbidity, 48% had two or more, and 14% had all three (Fig 4). Seventy-five percent of patients had at least one of these comorbidities within one year after ART initiation.

## Discussion

In this first study to determine incidence and predictors of new onset impaired fasting glucose, diabetes, overweight, and obesity following ART initiation in a longitudinal observational cohort in Latin America, we find a high incidence of these outcomes, most of which occurred within the 12 months following ART start. There was also a 13% prevalence of elevated CVD risk per the Framingham risk score, and a striking degree of overlap between cardiometabolic comorbidities, with the vast majority (79%) having at least one risk factor and almost half (48%) having two or more metabolic risks. These data have direct implications for HIV treatment programs in the region, and indicate that they should plan for an increase in metabolic and cardiac risk in many of those initiating ART.

The incidence of insulin resistance that we found in our cohort is concerning, as impaired fasting glucose was observed in almost half of our cohort in a span of two years, despite the fact that very few patients (13%) were receiving protease inhibitors, which are traditionally associated with this phenomenon,[12, 13, 29, 35] but not recommended for patients initiating ART in the DR. At the time of writing, a number of investigators have noted the rising prevalence of diabetes in LMIC,[16, 17] but only two studies have examined the incidence of diabetes in LMICs outside of Latin America: one in the Democratic Republic of Congo reported DM incidence of 9.8/1000 PYFU, and one in Thailand reporting DM incidence of 5/1000 PYFU.[36, 37] Other data from Latin America imply that the prevalence of diabetes and cardiovascular risk in people living with HIV is increasing; one cross-sectional study suggests high prevalence of DM in an aging population of HIV+ individuals, and an additional study in Brazil shows that the rate of increase in deaths attributable to CVD and DM is higher among HIV+ individuals compared to those without HIV.[16, 17]

The reported incidence of DM among HIV+ individuals in high income countries ranges from 4.42 to 47 cases per 1000 PYFU, placing the incidence seen in our cohort (28/1000 PYFU) at the high end of that seen in high income settings.[7, 13, 29, 35] Although prior studies have demonstrated an association between PI use and increased incidence of DM and dyslipidemia, [12, 13, 29, 35, 38, 39] we did not observe this association, though sample size of those exposed to PIs at any point was limited. It is possible that future increased access to integrase and strand transfer inhibitors in LMICs could further reduce the risk of IFG and DM.

Research has demonstrated rising prevalence of obesity in HIV+ cohorts in high income countries,[40] however, to our knowledge, no data on the incidence of obesity in observational HIV+ cohorts in Latin America exist. A randomized control trial of early ART and isoniazid treatment in Western Africa (ANRS 12136 Temprano Trial) demonstrated that obesity prevalence increased from 7.2% prior to ART start to 9.2%, and overweight prevalence rose from 20.4% to 24.8% after 24 months of ART.[19] Our data speak to the growing burden of obesity in cohorts initiating ART in Latin America, given the 45% prevalence of overweight/obesity seen in this cohort prior to ART start, and the 65% at the end of a median 2.9 years of observation. Recently published data from a sub-sample of the AIDS Clinical Trials Group PEARLS trial, which included data from 8 LMICs, 3 of which were in Latin America, show a rise in prevalence of overweight/obesity from 27% prior to ART initiation to 37% at 48 weeks after ART initiation.[20] Additionally, the weight gain seen in our cohort is unlikely solely attributable to food provision for HIV+ patients, as food provision is not a standard part of the Dominican national treatment program, nor was it available to cohort participants. The 24% obesity prevalence seen at the end of our study is comparable to cross sectional studies the U.S. and Brazil (17 to 32%).[41–49] The similar rates of obesity between this LMIC and more developed countries is particularly problematic given the limited access to primary care in the Dominican Republic, posing a greater challenge for controlling CVD risk in these patients. The association seen between BMI and dyslipidemia in our cohort implies that there are direct metabolic consequences for the weight gain observed in this cohort.

Using the Framingham CVD risk calculator to assess ten-year cardiovascular risk in the cohort, we found a 13% prevalence of elevated CVD risk. This is higher than other studies of HIV+ individuals in LMICs: 10.7% in Nigeria, 9.9% in Thailand, and 3.6% in Uganda, but comparable to the 11–18% prevalence found in a study of HIV negative individuals in Latin America.[23, 50–52] This is concerning as our cohort is slightly younger than the Latin American cohort (median ages 42.4 vs. 44.6), and the Framingham risk score is known to underestimate CVD risk in HIV+ individuals.[3, 53] Of the thirty participants who met criteria for hypertension in this sample, seventeen (57%) were uncontrolled, either meeting criteria for hypertension but not on treatment, or on treatment with last blood pressure >140/90 (130/80 for diabetics). Improving control of hypertension in this population could be an effective way to mitigate this elevated CVD risk. The high degree of overlap between IFG/DM, overweight/obesity, and dyslipidemia, with 48% of the cohort having two or more of these conditions, speaks to the burden of CVD risk within the cohort and parallels findings of a prior investigation which found an association between dyslipidemia and new-onset DM.[7]

Certain limitations should be noted. The ADA suggests using two FPG to diagnose DM, but a confirmatory measurement was not routinely available in this LMIC. However, prior studies have reported DM incidence using one FPG measurement.[27–29] Many lipid panels were not fractionated, which narrowed our definition for dyslipidemia and reduced the number of patients for whom we could calculate CVD risk scores. The prevalence of metabolic syndrome could not be assessed in this cohort because the definition would require waist circumference measurements or an assessment of visceral adipose tissue, fractionated lipid panels, and inflammatory biomarker data for all patients, which were not available. We did not collect family history of DM, or other CVD or risk factors. Information bias may affect our sample; we did not include those patients missing FPG or BMI measurements after initiation of ART, some of whom were lost to follow up and may have been more seriously ill. The small sample size did not provide enough power to undertake an analysis of the differential impact of ART drug type on metabolic parameters. Lastly, the follow up in this cohort is relatively short, with a median observation time of 2.9 years after ART initiation. Despite this limitation, we saw increases in our outcomes of interest that were clinically important.

Our findings of high incidence of diabetes and obesity after ART initiation have significant clinical implications for the management of care for HIV+ patients in Latin America. It is concerning that, after a median 24 months on ART, over half the cohort met criteria for IFG or DM, and half were overweight or obese. The burden of metabolic comorbidity is worrisome as a majority (87%) of these patients was receiving NNRTI-based regimens, which are less associated with insulin resistance than PI-based regimens. Many programs providing HIV care in LMICs, including those in the DR, are “silo-ed” into clinics focused on HIV treatment, and not integrated with primary care. Our findings demonstrate missed opportunities for lipid control, hypertension control, and lifestyle interventions that could reduce risk for obesity and DM. Given the rapid development of IFG/ DM and overweight/obesity after ART initiation, and the high prevalence of overlapping cardiometabolic risks in this cohort, we advocate for the integration of assessment and treatment of cardiovascular risk into HIV care.

This increased risk for CVD is particularly important in Latin American settings, as cardiovascular risk is highly prevalent in the general population and is a significant cause of mortality in the region.[21–23] If these risks are not addressed, gains in life expectancy from successful ART could be erased by morbidity and mortality from cardiovascular disease. Data regarding diabetes and obesity incidence in larger Latin American cohorts would be useful to define this risk. Innovative care models that incorporate cardiovascular risk reduction and non-communicable disease care into HIV treatment programs in LMICs are needed to prevent cardiovascular disease.

## Supporting Information

S1 Data Set Data Underlying Findings Reported in Manuscript. Variable definitions are included in second sheet.
(XLSX)
